# Enhancing sorafenib-mediated sensitization to gemcitabine in experimental pancreatic cancer through EMAP II

**DOI:** 10.1186/1756-9966-32-12

**Published:** 2013-03-06

**Authors:** Niranjan Awasthi, Changhua Zhang, Stefan Hinz, Margaret A Schwarz, Roderich E Schwarz

**Affiliations:** 1 Division of Surgical Oncology, Department of Surgery, The University of Texas Southwestern Medical Center, Dallas, TX 75390, USA; 2 Department of Pediatrics, The University of Texas Southwestern Medical Center, Dallas, TX 75390, USA; 3 Hamon Center for Therapeutic Oncology Research, Simmons Comprehensive Cancer Center, The University of Texas Southwestern Medical Center, Dallas, TX 75390, USA; 4 IU Health Goshen Center for Cancer Care, Professor of Surgery, Indiana University, 200 High Park Avenue, Goshen, IN 46526, USA

## Abstract

**Background:**

Pancreatic ductal adenocarcinoma (PDAC) is one of the most aggressive human malignancies and tends to be relatively resistant to conventional therapies. Activated Ras oncogene mutations are found in up to 90% of PDAC, leading to activation of the Ras/Raf/MEK/ERK signaling pathway. Sorafenib is a multikinase inhibitor of the Ras/Raf/MEK/ERK pathway and of tumor angiogenesis. Endothelial monocyte activating polypeptide II (EMAP) enhances gemcitabine effects in PDAC. Antitumor activity of sorafenib was evaluated in combination with gemcitabine (Gem) and the antiangiogenic agent EMAP in experimental PDAC.

**Methods:**

Cell proliferation and protein expression were analyzed by WST-1 assay and Western blotting. Animal survival studies were performed in murine PDAC xenografts.

**Results:**

Sorafenib decreased phospho-MEK, phospho-ERK1/2, phospho-p70S6K and phospho-4EBP-1 expression in PDAC cells. Sorafenib inhibited in vitro proliferation of all four PDAC cell lines tested. Additive effects on cell proliferation inhibition were observed in the gemcitabine-sorafenib combination in PDAC cells, and in combinations of sorafenib or EMAP with gemcitabine in endothelial (HUVEC) and fibroblast (WI-38) cells. Sorafenib, alone or in combination with gemcitabine and EMAP, induced apoptosis in HUVECs and WI-38 cells as observed via increased expression of cleaved poly (ADP-ribose) polymerase-1 (PARP-1) and caspase-3 proteins. Compared to controls (median survival: 22 days), animal survival increased after Gem therapy (29 days) but not in sorafenib (23 days) or EMAP therapy alone (25 days). Further increases in survival occurred in combination therapy groups Gem+sorafenib (30 days, p=0.004), Gem+EMAP (33 days, p=0.002), and Gem+sorafenib+EMAP (36 days, p=0.004), but not after the sorafenib+EMAP combination (24 days).

**Conclusions:**

These findings demonstrate that the addition of a polymechanistic antiangiogenic agent such as EMAP can enhance the combination treatment effects of sorafenib and cytotoxic PDAC therapy.

## Introduction

Pancreatic ductal adenocarcinoma (PDAC) remains a deadly human cancer with very poor prognosis and a 5-year survival of less than 5% [1]. This is primarily related to its late clinical presentation, early and aggressive local or metastatic progression and high resistance to conventional chemotherapy and radiation treatments. Gemcitabine (Gem), a cytotoxic nucleoside analog, is the most widely used single agent chemotherapeutic treatment for locally advanced and metastatic PDAC [2]. The efficacy of gemcitabine remains modest with a median survival of approximately 6 months and one-year survival of less than 20% [2-4]. Currently several clinical studies are underway to explore combination treatment benefits of gemcitabine with other cytotoxic, antiangiogenic or targeted agents for novel and more effective therapeutic strategies for PDAC. In addition, FOLFIRINOX is a combination cytotoxic regimen that has shown a somewhat greater efficacy but also greater toxicity potential compared to gemcitabine [5].

The K-ras oncogene is mutated in up to 90% of PDAC [6-8], leading to constitutive activation of the Ras/Raf/MEK/ERK signal transduction pathway and suggesting that this pathway could represent an important target for PDAC therapy. Sorafenib (So, Nexavar, BAY 43-9006) is a novel, potent, orally available multikinase inhibitor targeting Raf serine/threonine kinases as well as different receptor tyrosine kinases including vascular endothelial growth factor receptor (VEGFR), platelet derived growth factor receptor (PDGFR), c-Kit, FLT-3 and RET [9,10]. In preclinical studies sorafenib has shown significant antitumor responses in several tumor types including renal cell carcinoma, pancreatic cancer, colon cancer, breast cancer and melanoma based in part on its inhibitory effect on the Ras/Raf/MEK/ERK and angiogenesis pathways [9-11]. Sorafenib is approved for the clinical treatment of hepatocellular carcinoma and renal cell carcinoma [12]. A phase I trial of sorafenib plus gemcitabine in advanced PDAC showed that this combination was well tolerated and that 57% patients experienced stable disease [13]. More recently, a phase II trial of sorafenib plus gemcitabine showed no significant clinical activity in advanced PDAC [14]. These results support an evaluation of the addition of other antitumor agents to sorafenib plus gemcitabine for targeting multiple pathways that partake in PDAC progression.

Activated angiogenesis mechanisms are essential for the progression of primary and metastatic solid tumors including PDAC. Antiangiogenic agents including bevacizumab, an antibody against vascular endothelial growth factor (VEGF) [15,16], the matrix metalloproteinase inhibitor marimastat [17], the cyclooxygenase-2 inhibitor celecoxib [18] and various other TKIs [19] have been tested clinically in PDAC with limited survival benefit [20]. Endothelial monocyte activating polypeptide II (EMAP, E) is a proinflammatory cytokine with antiangiogenic and antiendothelial activities. Although EMAP has no effect on in vitro AsPC-1 PDAC cell line proliferation or apoptosis [21,22], it has potent effects on endothelial cells (ECs) such as inhibition of proliferation, migration and vascularization as well as induction of apoptosis [23,24]. EMAP has been shown to suppress primary and metastatic tumor growth [23,25,26] that could be related to its ability to bind VEGF receptors and α5β1 integrin, leading to interference in fibronectin- and VEGF signaling [27,28]. EMAP has recently been shown to improve gemcitabine and docetaxel response in experimental PDAC [21,29,30]. In the present study, we tested the hypothesis that combination treatment of EMAP with sorafenib and gemcitabine can enhance antitumor effects by blocking multiple critical pathways leading to progression of PDAC, to define an option for future PDAC clinical applications.

## Materials and methods

### Materials

Gemcitabine was purchased from Eli Lilly (Indianapolis, IN). Sorafenib was purchased from LC Laboratories, Inc. (Woburn, MA). Recombinant human EMAP was prepared as previously described [31], and the cell proliferation reagent WST-1 was purchased from Roche Diagnostic Corporation (Indianapolis, IN).

### Cell culture

The human pancreatic cancer cell line AsPC-1, human umbilical vein endothelial cells (HUVECs) and human fibroblast cell line WI-38 were all purchased from the American Type Culture Collection (ATCC, Rockville, MD). AsPC-1 and WI-38 cells were grown in RPMI 1640 medium and DMEM, respectively (Sigma Chemical Co. St. Louis, MO) supplemented with 10% fetal bovine serum (FBS). HUVECs were grown in EndoGRO-LS medium containing endothelial cell growth supplements (Millipore Corp., Billerica, MA).

### Cell viability assay

In vitro cell viability was evaluated by using WST-1 reagent as per the manufacturer’s instructions. Briefly, four thousand cells were plated in a 96-well plate and after 16 hours the medium was replaced with low serum medium. Cells were treated with gemcitabine, sorafenib and EMAP. The range of concentrations used for gemcitabine, sorafenib and EMAP were from 100 nM to 10 μM. After a 72-hour incubation, WST-1 reagent (10 μl) was added in each well and after 2 hours absorbance was measured at 450 nm using a microplate reader.

### Western blot analysis

Cell monolayers were treated with gemcitabine (10 μM), sorafenib (10 μM) or EMAP (10 μM) and incubated for 16 hours. Total cell lysates were prepared, and equal amounts of protein were separated by SDS-PAGE and transferred to PVDF membranes (Bio-Rad, Hercules, CA). The membranes were blocked for 1 hour in blocking solution (5% milk in TBS-T [Tris-buffered saline containing Tween-20]) and incubated overnight at 4°C with the following antibodies: phospho-MEK (Ser221), total-MEK, phospho-ERK1/2 (Thr202/Tyr204), total-ERK1/2, phospho-p70 S6 kinase (Thr389), total-p70 S6 kinase, phospho-4E-BP1 (Thr37/46), Total-4E-BP1, cleaved poly (ADP-ribose) polymerase-1 (PARP-1), cleaved caspase-3 (all from Cell Signaling Technology, Beverly, MA) or α-tubulin (Sigma). After primary antibody incubation, the membranes were incubated for 1 hour with corresponding HRP-conjugated secondary antibodies (Pierce Biotechnologies, Santa Cruz, CA). Protein bands were detected using ECL reagent (Perkin Elmer Life Sciences, Boston, MA) on autoradiographic film and quantitated by densitometry.

### Animal survival analysis

All animal procedures were performed according to the guidelines and approved protocols of the University of Texas Southwestern Medical Center (Dallas, TX) Institutional Animal Care and Use Committee (Animal Protocol Number 2008-0348). Animal survival studies were performed using 6- to 8-week-old female SCID mice, as previously described [32]. Briefly, mice were intraperitoneally injected with AsPC-1 cells (0.75x10^6^), after two weeks mice were randomly grouped (n=6 to 8 per group) and treated intraperitoneally with PBS (control), gemcitabine (100 mg/kg, twice per week), sorafenib (30 mg/kg, 5 times per week) or EMAP (80 μg/kg, 5 times per week) for next two weeks. Animals were euthanized when appeared moribund according to predefined criteria including rapid body weight gain or loss (>15%), tumor size, lethargy, inability to remain upright and lack of strength. Animal survival was evaluated from the start of therapy until death. Two mice (one each from Gem+E and Gem+So+E groups) were removed from the study during the treatment period due to early development of severe toxicity.

### Statistical analysis

*In vitro* cell proliferation assay and Western blot densitometric analysis results are expressed as mean ± standard deviation (SD). Statistical significance was analyzed by the two-tailed Student’s t-test using GraphPad Prism 4 Software (GraphPad Software, San Diego, CA). Statistical differences in animal survival studies were analyzed with StatView for Macintosh version 5.0.1 (SAS, Carey, NC) by nonparametric survival statistics and logrank testing. P values of <0.05 were considered to represent statistically significant group differences.

## Results

### Effect of sorafenib on Ras/Raf/MEK/ERK signaling

**Figure 1:**
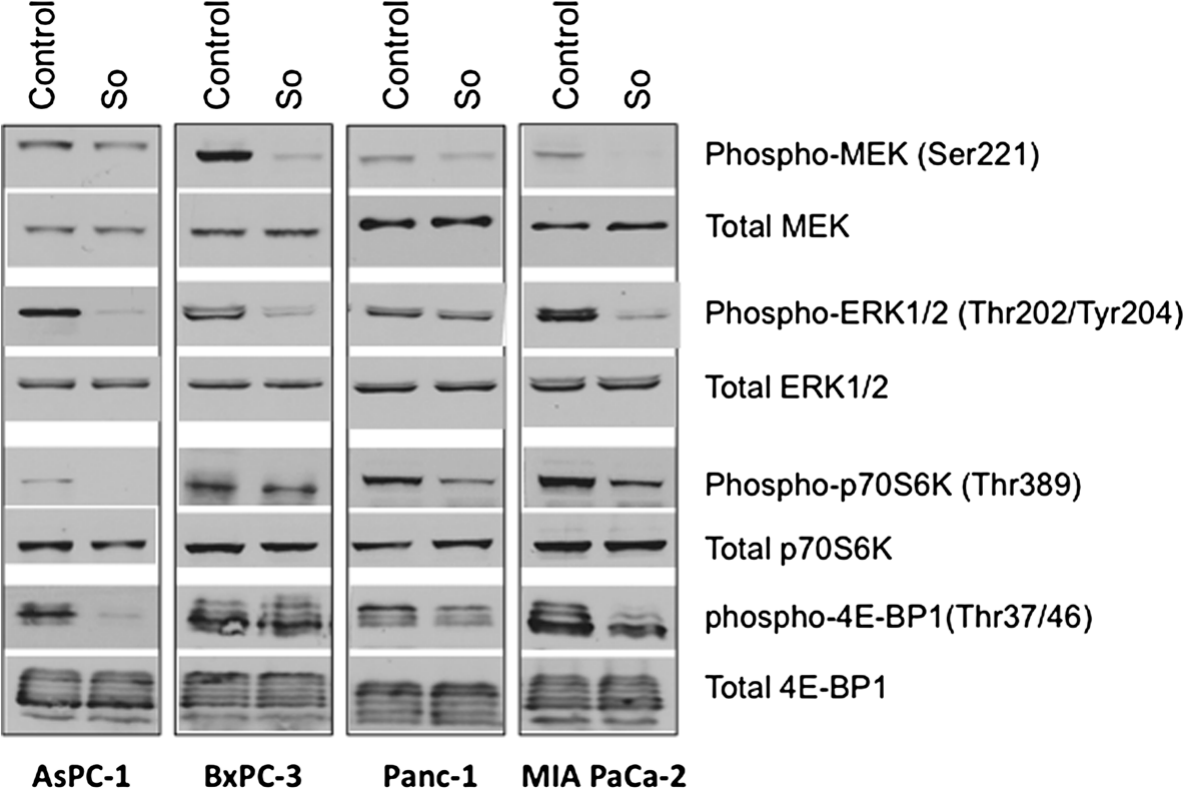
**Sorafenib inhibits the Raf/MEK/ERK signaling pathway.** Human PDAC cells (AsPC-1, BxPC-3, Panc-1, MIA PaCa-2) were treated with sorafenib (So) (10 μM) for 4 hours. Total cell extracts were analyzed by immunoblotting for p-MEK (Ser221), total MEK, p-ERK1/2 (Thr202/Tyr204), total ERK, p-p70 S6K (Thr389), total p70 S6K, p-4E-BP1 and total 4E-BP1 proteins. Data are representative of two independent experiments with similar results.

Evaluation of the sorafenib effect on the Ras/Raf/MEK/ERK signaling pathway in human PDAC cell lines revealed that 4-hour sorafenib treatment (10 μM) caused a significant decrease in the expression of phospho-MEK (Ser221), phospho-ERK1/2 (Thr202/Tyr204) and the downstream signaling proteins phospho-p70 S6 kinase (Thr389) and phospho-4E-BP1 (Thr37/46) in AsPC-1, Panc-1 and MIA PaCa-2 cells (Figure 1). In BxPC-3 cells, sorafenib caused significant decrease in phospho-MEK and phospho-ERK but no significant change in downstream signaling proteins phospho-p70S6K and phospho-4E-BP1 (Figure 1). In the present study, we evaluated the effect of sorafenib on phospho-p-70S6K and phospho-4E-BP1 as these proteins have recently been shown to be downstream effectors of both AKT/mTOR and MEK/ERK signaling cascades [33].

### Effect of gemcitabine and sorafenib on PDAC cell proliferation

**Figure 2:**
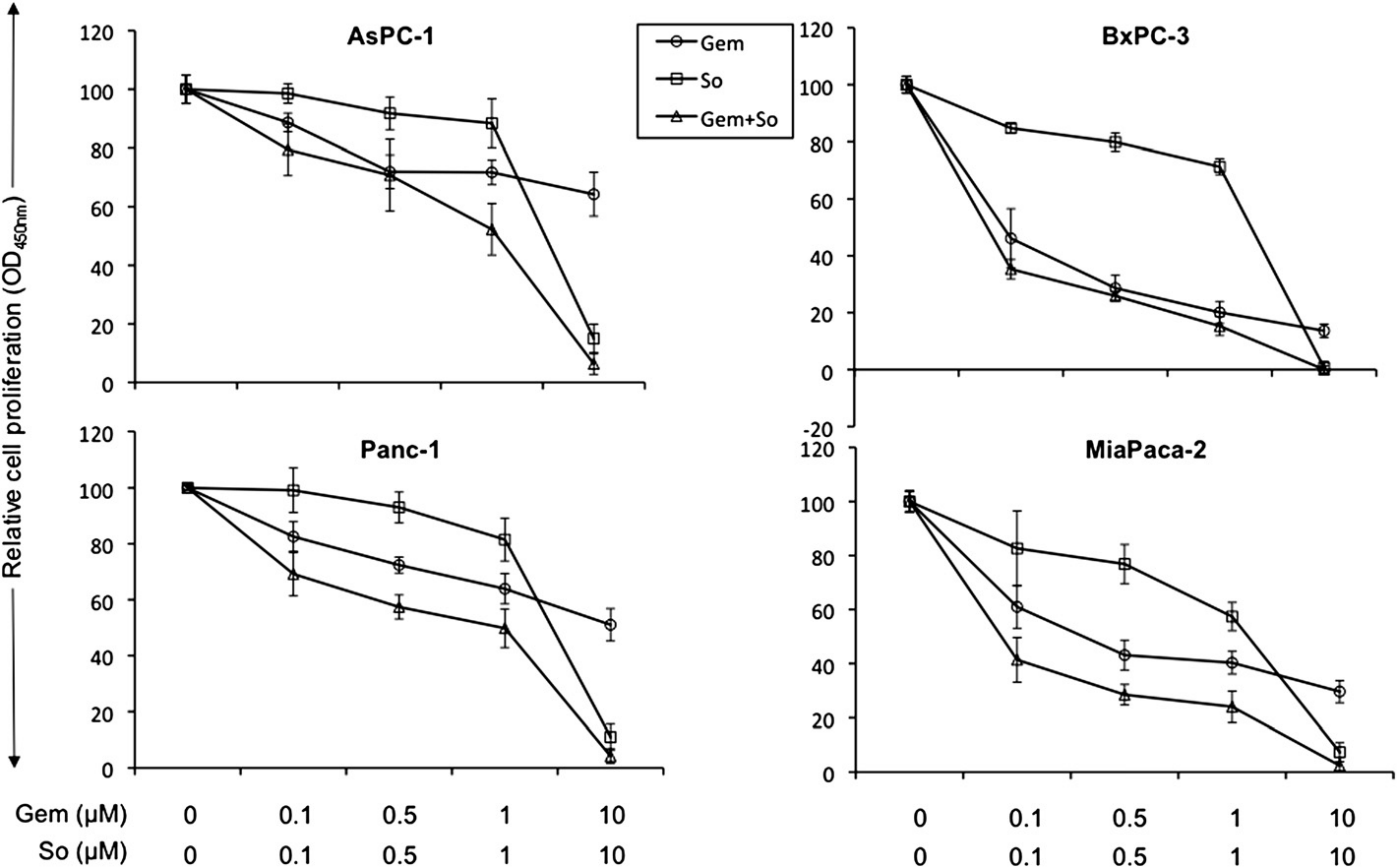
**Gemcitabine (Gem) and sorafenib (So) inhibit in vitro cell proliferation of PDAC cells.** AsPC-1, BxPC-3, Panc-1 and MIA PaCa-2 cells were plated on 96-well plates and treated with gemcitabine and sorafenib. After 72 hours, 10 μl WST-1 reagent was added in each well and incubated for 2 additional hours. The absorbance at 450 nm was measured using a microplate reader. The resulting number of viable cells was calculated by measuring absorbance of color produced in each well. Data are the mean ± SD of triplicate determinations.

In vitro cell proliferation analysis of PDAC cells showed that gemcitabine and sorafenib both inhibited PDAC cell line proliferation but had differential inhibitory effects. At 10 μM concentration of gemcitabine, percent inhibition in cell proliferation was 36, 86, 49 and 70 in AsPC-1, BxPC-3, Panc-1 and MIA PaCa-2 cells, respectively. At 10 μM concentration of sorafenib, percent inhibition in cell proliferation was 85, 99, 89 and 93 in AsPC-1, BxPC-3, Panc-1 and MIA PaCa-2. The combination of gemcitabine and sorafenib had stronger inhibitory effects on the proliferation of all four PDAC cells at almost all concentrations tested (Figure 2). A relatively greater inhibitory effect of combination treatment on PDAC proliferation was more obvious at lower concentrations. Percent inhibition in cell proliferation after 100 nM gemcitabine was 11, 54, 17 and 39, after 100 nM sorafenib 1, 15, 1 and 17, and after combination of these two agents 21, 65, 31 and 59 in AsPC-1, BxPC-3, Panc-1 and MIA PaCa-2, respectively (Figure 2).

### Effect of gemcitabine, sorafenib and EMAP on EC and fibroblast proliferation

**Figure 3:**
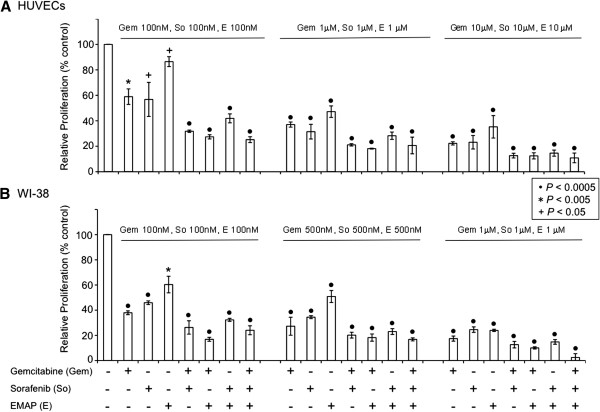
**Gemcitabine (Gem), sorafenib (So) and EMAP (E) inhibit in vitro cell proliferation of EC (HUVECs) and fibroblast cells (WI-38).** Cells were plated on 96-well plate and treated with gemcitabine, sorafenib and EMAP. After 72 hours incubation, WST-1 reagent was added in each well and number of viable cells was calculated by measuring absorbance of color produced in each well. Data are representative of mean values ± SD of triplicate determinants. Symbols +, * and • represent p values of less than 0.05, 0.005 and 0.0005 compared to controls.

Targeting endothelial cells and fibroblasts for solid tumor treatment has been shown to be potentially quite effective [34,35]. In our study, analysis of *in vitro* HUVEC and WI-38 cell proliferation in growth factor containing medium revealed that single agent gemcitabine, sorafenib and EMAP induced significant dose-dependent inhibitory effects. Importantly, combination of these agents had some additive effects on inhibition of cell proliferation of both cell lines. At an intermediate concentration of gemcitabine (1 μM), sorafenib (1 μM) and EMAP (1 μM), the percent inhibition in HUVEC proliferation was 63, 69, 53, 79, 82, 72 and 79 in the Gem, So, EMAP, Gem+So, Gem+EMAP, So+EMAP and Gem+So+EMAP groups, respectively. In fibroblast WI-38 cells at an intermediate concentration of gemcitabine (500 nM), sorafenib (500 nM) and EMAP (500 nM) the percent inhibition in WI-38 proliferation was 73, 66, 49, 80, 82, 77 and 83 in the Gem, So, EMAP, Gem+So, Gem+EMAP, So+EMAP and Gem+So+EMAP groups, respectively (Figure 3).

### Effect of gemcitabine, sorafenib and EMAP on apoptosis markers

**Figure 4:**
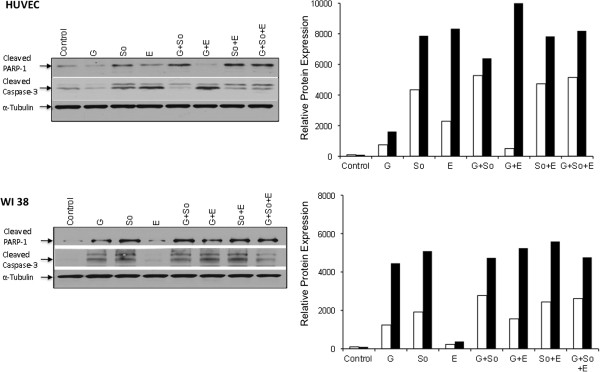
**Effects of gemcitabine (G), sorafenib (So) and EMAP (E) treatment on cleavage of PARP-1 and caspase-3 proteins.** A sub-confluent cell monolayer was treated with gemcitabine (10 μM), sorafenib (10 μM) and EMAP (10 μM). After 16 hours of incubation, total cell lysate was prepared and analyzed by immunoblotting for cleaved PARP-1, cleaved caspase-3 and α-tubulin (loading control) proteins. The intensity of bands was quantitated by densitometry and is represented as the bar graph for cleaved PARP-1 (open bar) and cleaved caspase-3 (closed bar) after normalizing against α-tubulin expression. Data are representative of two independent experiments with similar results.

Western blot analysis to evaluate if inhibition in cell proliferation was due to the induction in apoptosis revealed that sorafenib treatment either alone or in combination with gemcitabine and EMAP induced apoptosis as observed via PARP-1 cleavage and caspase-3 cleavage in HUVECs and WI-38 cells (Figure 4). Sorafenib-induced expression of cleaved PARP-1 and cleaved caspase-3 was similar in HUVECs and WI-38 cells. Gemcitabine caused a significant increase in PARP-1 or caspase-3 cleavage in WI-38 fibroblast cells but no detectable change in HUVECs (Figure 4). EMAP treatment caused a small change in these apoptosis marker protein in HUVECs but not in WI-38 cells. In a parallel setting with AsPC-1 PDAC cells, no detectable change in apoptosis marker proteins was observed after gemcitabine, sorafenib or EMAP treatment (data not shown).

### Effect of gemcitabine, sorafenib and EMAP on animal survival

**Figure 5:**
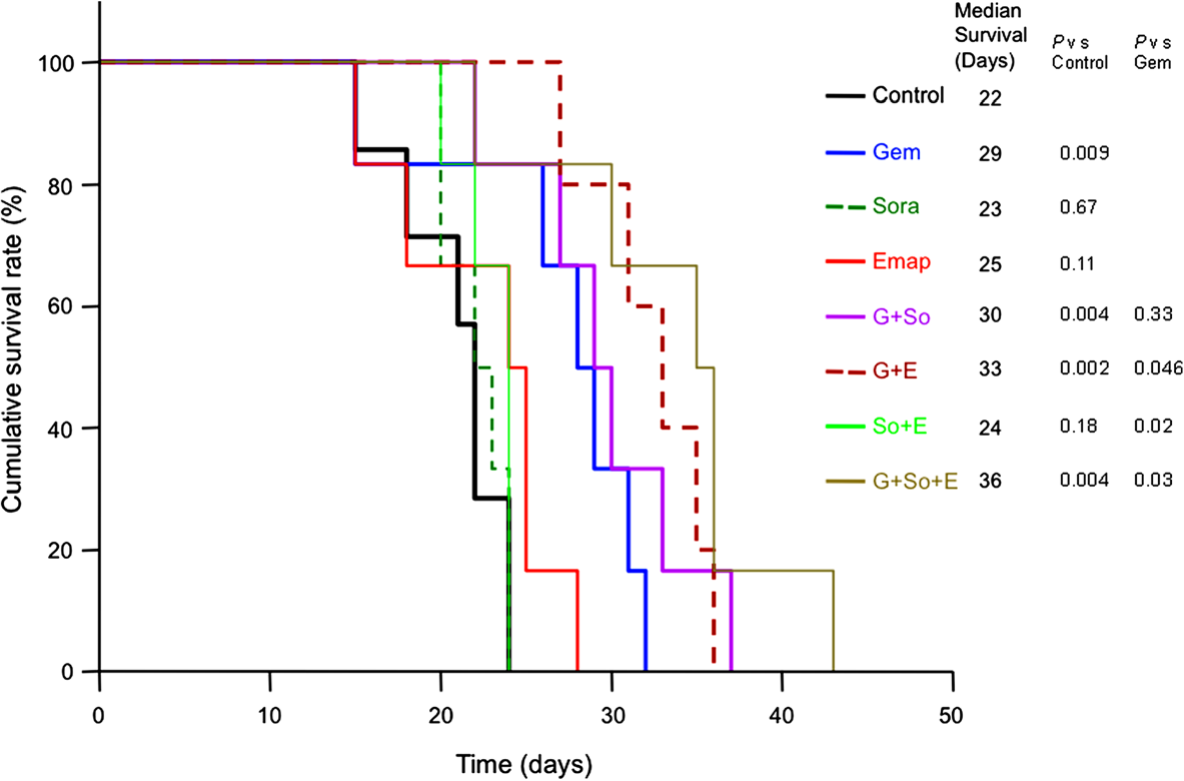
**Effects of gemcitabine (Gem), sorafenib (So) and EMAP (E) treatment on the overall survival of mice.** AsPC-1 cells (0.75 x 10^6^) were injected intraperitoneally in SCID mice and treatment started after 2 weeks with gemcitabine (100 mg/Kg, 2 times a week), sorafenib (30 mg/Kg, 5 times a week), and EMAP (80 μg/Kg, 5 times a week) for 2 weeks. The curve represents the survival time from the beginning of therapy.

*In vivo* animal survival studies in SCID-NOD mice resulted in a median survival (m.s.) of 22 days in the control group without treatment. Median animal survival was increased significantly after Gem (29 days, p=0.009 vs. control) but not after sorafenib (23 days, p=0.67 vs. control) or EMAP (25 days, p=0.11) monotherapy (Figure 5). Further improvement in animal survival was encountered in the combination therapy groups Gem+So (m.s. 30 days, p=0.004 vs. controls), Gem+EMAP (m.s. 33 days, p=0.002 vs. controls) and Gem+So+EMAP (m.s. 36 days, p=0.004 vs. controls). Compared to the Gem monotherapy group, median survival was significantly higher in the Gem+EMAP (p=0.046) and Gem+So+EMAP therapy group (p=0.03) but not in the Gem+So therapy group (p=0.3). Survival in the So+EMAP therapy group (m.s. 24 days, p=0.18 vs. control) was not significantly different from controls or single agent therapy groups (Figure 5). No sign of drug-related toxicity was observed in any of the treatment groups.

## Discussion

PDAC shows limited susceptibility to almost all classes of cytotoxic drugs. Several molecular genetic abnormalities in PDAC are being encountered with a high frequency, including activating K-ras mutation, loss of p16, p53 and DPC4 (deleted in pancreatic cancer, locus 4) function, and over-expression of multiple receptor tyrosine kinases [36,37]. Tumor heterogeneity resulting from the diverse molecular abnormalities acquired during malignant transformation creates a rationale to evaluate multi-targeted therapeutic strategies against many human malignancies including PDAC. Sorafenib is a novel, potent, small molecular mass inhibitor with combined anticancer activities through the inhibition of tumor cell proliferation and tumor angiogenesis. Combining conventional cytotoxic drugs, such as gemcitabine, with targeted agents that specifically interfere with key operational pathways responsible for PDAC progression, such as sorafenib, is gaining more traction in the efforts to identify more effective combination treatments for PDAC.

In PDAC progression, angiogenesis plays a critical role that is highly dependent on the complex interaction among tumor cells, ECs, immune cells, fibroblasts and other stromal components, all contributing to the well-characterized extensively desmoplastic and hypoxic local tumor microenvironment of pancreatic cancer. Specifically for this reason, antiendothelial and antiangiogenic agents may be beneficial in combination therapy approaches for PDAC treatment. In the present study we evaluated the antitumor activity of sorafenib, and the enhancement of gemcitabine response by addition of sorafenib and the antiangiogenic agent EMAP in experimental pancreatic cancer. We demonstrate that in PDAC cells sorafenib treatment effectively blocked phosphorylation of MEK (Ser221), ERK1/2 (Thr202/Tyr204) and downstream target proteins phospho-p70 S6K (Thr389) and phospho-4E-BP1 (Thr37/46) in most of the cell lines tested except BxPC-3, where upstream MEK and ERK phosphorylation was inhibited but not the downstream signaling proteins p70S6K or 4-EBP-1. These findings suggest that sorafenib may cause some specific effects that result in blockage of Ras/Raf/MEK/ERK signaling and interfere with pancreatic cancer cell proliferation, differentiation and survival. Sorafenib treatment decreased cell proliferation and induced apoptosis in ECs and fibroblasts indicating that the in vivo antitumor effects of sorafenib may be due to its direct cytotoxic effects on various tumor cellular components, in addition to its antiangiogenic properties.

Previous studies have shown marked heterogeneity in gemcitabine and other chemotherapeutic agent response towards PADC cells [38-40]. We also observed a heterogeneous response of sorafenib and gemcitabine in inhibiting cell proliferation of four PDAC lines tested. Both agents caused inhibition of cell proliferation to different extents and the addition of sorafenib improved gemcitabine effects. Effects of combinations of EMAP with sorafenib and gemcitabine were evaluated in ECs and fibroblast cells, and a significant additive effect on inhibition of cell proliferation was observed compared with single or dual agent treatment. A gemcitabine plus sorafenib combination was found to be effective in preclinical and phase I trials of PDAC, lending support to the importance of combining cytotoxic drugs with agents inhibiting Ras/Raf/MEK/ERK pathways and angiogenesis [9-11,13]. However, a phase II trial showed no meaningful effect of the gemcitabine plus sorafenib combination in advanced PDAC patients [14]. The very small number of 17 patients and 94% of patients carrying metastatic disease were the contributing factors in the negative phase II clinical trial results [14]. These results also indicate the importance of targeting other relevant pathways that contribute in the progression of PDAC. Currently, two phase II trials are evaluating the combination treatment benefits of gemcitabine, sorafenib and the EGFR inhibitor erlotinib in advanced PDAC. The anti-vascular endothelial growth factor agent bevacizumab, the first FDA-approved angiogenesis inhibitor, showed promising phase II data in combination with gemcitabine in PDAC patients but failed to demonstrate any survival benefit in phase III trials [41]. Since sorafenib inhibits the raf kinase and VEGF pathways, we assumed that the addition of EMAP, an inhibitor of VEGF and integrin-fibronectin pathways [25,27], to gemcitabine and sorafenib would potentially improve in vivo outcome of clinical PDAC. This assumption was based on the effective in vitro combination data with EMAP in previous studies showing EMAP enhancing antitumor effects of gemcitabine paired with bevacizumab [21] or with the mTOR and AKT inhibitor NVP-BEZ235 [40].

Activating K-ras mutations are highly prevalent and have been shown to be important in the initiation and progression of pancreatic cancer. Farnesyltransferase inhibitors that can block K-ras activation have been tested clinically, but the results showed insufficient antitumor activity perhaps indicating the importance of multi-targeted strategies against PDAC that can extend beyond the inhibition of a single upstream mediator within a frequently activated signaling pathway [42]. Later studies focused on therapeutic targeting of the Ras/Raf/MEK/ERK network in combination with other important molecular targets by multikinase inhibitors such as sorafenib that has been shown to generate some antitumor activity as single agent in a pancreatic cancer cells [43]. Our results not only corroborate with these findings, but also demonstrate the impact of sorafenib and its combinations with gemcitabine on several other, potentially relevant cell types and on experimental PDAC survival. In addition, we tested combination treatment benefits of sorafenib with gemcitabine and EMAP, based on previous studies in our lab that showed EMAP-derived improvements of gemcitabine effects in vivo [29,31]. The observed advantages of combining these agents can be interpreted as supportive of a rationale to a multi-agent clinical approach to PDAC that includes a multikinase inhibitor, a targeted multi-pathway blocker such as sorafenib, and an antiendothelial or antiangiogenic agent. Although optimal combination conditions and exact mechanisms are still not clear, these findings may provide a solid foundation for future evaluation of combination benefits of agents displaying these known effects.

Based on the limited efficacy of sorafenib in a therapeutic approach confined to 2 weeks, prolonged or intermittent dosing could be considered as an option for achieving progression-free benefits more likely. While we have not tested this approach in our experiments to date, there is concern over the true ability to obtain superior antitumor effects in the long term. Other than the commonly known side effects that could prevent this from being a clinically feasible strategy, persistent long term use of sorafenib might also lead to the development of resistant tumor cells with a more aggressive phenotype due to some epithelial-to-mesenchymal transition (EMT) at the time of tumor recurrence [44]. Therefore an altered/decreased dose of a multikinase inhibitor such as sorafenib in combination with a chemotherapeutic and antiangiogenic/targeted agent may provide a better therapeutic option.

In summary, our present study demonstrates that the multikinase inhibitor sorafenib, either alone or in combination with gemcitabine and EMAP, induced strong antiproliferative and proapoptotic effects in vitro. While the in vivo effects of sorafenib were limited, the addition of EMAP enhanced the combination treatment of sorafenib and gemcitabine in improving animal survival. This provides evidence that targeting multiple mechanisms of pancreatic cancer progression can be a promising therapeutic approach for PDAC treatment.

## Competing interests

The authors declare that they have no competing interests

## Authors’ contribution

NA was involved in the design of the study, execution of the experiments, data analysis and drafting the manuscript. CZ and MAS participated in the animal survival studies. SH participated in the Western blot analysis. RES conceived of the study, and was involved in the planning and design of the study, data analysis and drafting of the manuscript. All the authors read and approved the manuscript.
